# Identification and Overexpression of a Knotted1-Like Transcription Factor in Switchgrass (*Panicum virgatum* L.) for Lignocellulosic Feedstock Improvement

**DOI:** 10.3389/fpls.2016.00520

**Published:** 2016-04-28

**Authors:** Wegi A. Wuddineh, Mitra Mazarei, Ji-Yi Zhang, Geoffrey B. Turner, Robert W. Sykes, Stephen R. Decker, Mark F. Davis, Michael K. Udvardi, C. Neal Stewart

**Affiliations:** ^1^Department of Plant Sciences, University of TennesseeKnoxville, TN, USA; ^2^BioEnergy Science Center, Oak Ridge National LaboratoryOak Ridge, TN, USA; ^3^Plant Biology Division, Samuel Roberts Noble FoundationArdmore, OK, USA; ^4^National Renewable Energy Laboratory, GoldenCO, USA

**Keywords:** gene regulation, lignin, gibberellin, cell walls, cellulose, hemicellulose, sugar release, biofuel

## Abstract

High biomass production and wide adaptation has made switchgrass (*Panicum virgatum* L.) an important candidate lignocellulosic bioenergy crop. One major limitation of this and other lignocellulosic feedstocks is the recalcitrance of complex carbohydrates to hydrolysis for conversion to biofuels. Lignin is the major contributor to recalcitrance as it limits the accessibility of cell wall carbohydrates to enzymatic breakdown into fermentable sugars. Therefore, genetic manipulation of the lignin biosynthesis pathway is one strategy to reduce recalcitrance. Here, we identified a switchgrass Knotted1 transcription factor, PvKN1, with the aim of genetically engineering switchgrass for reduced biomass recalcitrance for biofuel production. Gene expression of the endogenous *PvKN1* gene was observed to be highest in young inflorescences and stems. Ectopic overexpression of *PvKN1* in switchgrass altered growth, especially in early developmental stages. Transgenic lines had reduced expression of most lignin biosynthetic genes accompanied by a reduction in lignin content suggesting the involvement of PvKN1 in the broad regulation of the lignin biosynthesis pathway. Moreover, the reduced expression of the *Gibberellin 20-oxidase* (*GA20ox*) gene in tandem with the increased expression of *Gibberellin 2-oxidase* (*GA2ox*) genes in transgenic *PvKN1* lines suggest that PvKN1 may exert regulatory effects via modulation of GA signaling. Furthermore, overexpression of *PvKN1* altered the expression of cellulose and hemicellulose biosynthetic genes and increased sugar release efficiency in transgenic lines. Our results demonstrated that switchgrass PvKN1 is a putative ortholog of maize KN1 that is linked to plant lignification and cell wall and development traits as a major regulatory gene. Therefore, targeted overexpression of *PvKN1* in bioenergy feedstocks may provide one feasible strategy for reducing biomass recalcitrance and simultaneously improving plant growth characteristics.

## Introduction

Switchgrass, a C_4_ perennial prairie forage grass indigenous to North American grasslands, is a leading candidate as a lignocellulosic biofuel feedstock owing to its high yield of biomass, stress resistance, high nutrient-use efficiency, and its ability to thrive on marginal land unsuitable for row crops (Yuan et al., 2008). Controlled field studies to determine the net energy efficiency of switchgrass monoculture on marginal lands showed that it can produce as much as 540% more renewable energy than the non-renewable energy consumed for its production (Schmer et al., 2008), highlighting the potential of this feedstock as a renewable and clean energy source. One of the major obstacles for the development of this and other lignocellulosic biomass feedstocks for biofuels is biomass recalcitrance (resistance of the cellulose and hemicellulose in cell walls to breakdown into fermentable sugars). Currently, biomass must be pretreated before saccharification and fermentation, which is expensive (Ragauskas et al., 2006). Reducing recalcitrance to enhance the economic competitiveness of lignocellulosic-based biofuels is an overarching goal in bioenergy research (Lynd et al., 2008).

Lignin is a complex aromatic polymer composed of hydroxyphenyl, guaiacyl, and syringyl monolignols. Transgenic approaches have been used to reduce lignin, one of the major contributors to biomass recalcitrance. Mostly, a gene-by-gene strategy has been used to downregulate individual biosynthetic genes to reduce lignin (Fu et al., 2011a,b; Saathoff et al., 2011; Xu et al., 2011; Baxter et al., 2014). Several enzymes responsible for monolignol biosynthesis have been identified and functionally characterized in switchgrass (Escamilla-Trevino et al., 2010; Fu et al., 2011a,b; Saathoff et al., 2011; Xu et al., 2011; Shen et al., 2013a). Genetically targeting individual lignin biosynthesis genes to block specific branches in the lignin biosynthesis pathway has been successful, but this strategy appears to have limitations as the build-up of low molecular weight phenolic compounds and other fermentation inhibitors may eventually reduce biofuel output (Tschaplinski et al., 2012). The use of transcriptional repressors of the lignin biosynthesis pathway might be a more effective strategy, as shown by the recent work in which the switchgrass gene coding for MYB4 TF was overexpressed (Shen et al., 2012, 2013b).

Lignin biosynthesis is regulated by multi-layered network of TFs including top-tier NAC and second-tier MYB master regulators (Handakumbura and Hazen, 2012; Zhong and Ye, 2014). The NAC TFs, which activate the lignin biosynthesis pathway via activation of downstream MYB TFs have been well documented in dicots such as *Arabidopsis* and *Populus* (Zhao Q. et al., 2010; Zhong and Ye, 2014). Recent studies reported the identification of some rice and maize SWN that are functional orthologs of *Arabidopsis* SWNs (Zhong et al., 2011; Chai et al., 2015). Similarly, several second-tier MYBs that control the activation of downstream MYB TFs specific to the lignin biosynthesis pathway have been characterized. These include: the *Arabidopsis* MYB46/83 and its homologs in *Populus* (PtrMYB2/3/20/21), pine (PtMYB4/8), *Eucalyptus* (EgMYB2), rice (OsMYB46), and maize (ZmMYB46) (Zhong et al., 2011; Zhong and Ye, 2014). The lignin-specific MYBs act either as a repressors or activators of lignin biosynthesis pathway genes. Several MYB TFs that are repressors of the lignin biosynthesis pathway have been identified in dicots including *Arabidopsis* (MYB4/7/32) and *Eucalyptus* (MYB1) (Fornale et al., 2010; Shen et al., 2012; Zhong and Ye, 2014). MYB TFs that are activators of the lignin biosynthesis pathway were also identified in several dicots including *Arabidopsis* (MYB58/63 and MYB85), *Populus* (MYB26/28/90/152) and pine (MYB1) (Zhou et al., 2009; Zhong and Ye, 2014). However, with the exception of maize MYB31 (Fornale et al., 2010), wheat MYB4 (Ma et al., 2011) and a recently characterized switchgrass MYB4 TFs (Shen et al., 2012, 2013b), there is limited available information about the transcriptional regulation of lignin biosynthesis in monocots (Handakumbura and Hazen, 2012).

Knotted1(KN1)-like homeobox (KNOX) proteins are members of plant-specific TALE family of TFs, which play a crucial role in the maintenance of meristem tissues and the regulation of various morphogenic processes throughout plant development (Hay and Tsiantis, 2010). These TFs are grouped into two major classes based on their HD identity, intron positions and expression patterns (Kerstetter et al., 1994; Mukherjee et al., 2009; Hay and Tsiantis, 2010; Testone et al., 2012). Class I *KNOX* genes are mainly expressed in the SAM where they redundantly regulate stem cell maintenance, and in vascular cambium. Class II *KNOX* genes are diversely expressed and their functions are not as well characterized (Jackson et al., 1994; Hake et al., 2004; Du et al., 2009). Members of class I *KNOX* genes from *Arabidopsis* (*KNAT1* also known as *BREVIPEDICELLUS/BP*), peach (*KNOPE1*), *Populus* (*ARBORKNOX2/ARK2*), and maize (*KN1*) (Mele et al., 2003; Du et al., 2009; Testone et al., 2012; Townsley et al., 2013), and a member of class II *KNOX* genes from *Arabidopsis* (*KNAT7*; Li et al., 2012) have been shown to function as repressors of lignin biosynthetic genes. *KNAT1* overexpression in *Arabidopsis* has been shown to decrease lignin deposition as well as the expression of most lignin biosynthetic genes while binding the promoters of at least two lignin biosynthetic genes, *COMT* and *CCoAOMT* (Mele et al., 2003). Similarly, a *Populus* ortholog of KNAT1, ARK2 has also been shown to negatively regulate several genes in the lignin biosynthesis pathway followed by marked reduction of the polymerized lignin in the stem (Du et al., 2009). Interestingly, *ARK2* overexpression in *Populus* was found to be associated with reduced expression of secondary cell wall cellulose synthase genes (Ces) including three xylem-specific (*PttCesA1*, *PttCesA3*-1, and *PttCesA3*-2) and two phloem-specific (*PttCesA2* and *estExt_Genewise1_v1.C_LG_VI2188*) *CesA* genes (Du et al., 2009). Despite vast information on class I *KNOX* gene regulation of cell wall biosynthesis pathway in dicots, such reports are limited in monocots with the exception of maize.

A recent study showed that among three members of maize class I *KNOX* genes investigated for roles in the regulation of lignin biosynthesis, overexpression of *KN1* significantly reduced lignin deposition in maize and tobacco while altering the expression of only two of the four lignin biosynthetic genes analyzed in tobacco (Townsley et al., 2013). However, it was not reported whether similar changes in the expression of lignin biosynthetic genes were observed in maize. This gene has also been shown to regulate gibberellin (GA) signaling via the positive transcriptional regulation of maize *GA 2-oxidase* (*GA2ox1*; codes for an enzyme that inactivates bioactive GA) (Bolduc and Hake, 2009). Overexpression of *KNAT1* has previously been shown to decrease the levels of *GA 20-oxidase* (*GA20ox1*) mRNA in *Arabidopsis* leaves (Hay et al., 2002) as did the overexpression of *ARK2* gene in hybrid aspen (*P. alba* ×*P. tremula*; Du et al., 2009). However, it remains to be determined whether *KNOX* genes regulate GA biosynthetic genes in monocots. GA signaling plays vital roles in the regulation of various developmental and growth processes and its downregulation has been shown to alter plant architecture and lignin deposition (Biemelt et al., 2004; Thomas et al., 2005; Zhao X.Y. et al., 2010). Our recent study demonstrated that the overexpression of the switchgrass GA catabolic gene *PvGA2ox5* caused reduced lignification and enhanced sugar release efficiency (Wuddineh et al., 2015). It appears, at least in model dicots, that KNOX TFs may regulate the lignification process via modulation of the GA signaling pathways. However, to the best of our knowledge, there are no published reports on whether class I KNOX TFs from monocots regulate the lignification process and any mechanism thereof.

With the exception of maize KN1-induced alteration of plant lignification, the potential of KNOX TFs to alter various growth and biomass characteristics of bioenergy crops is largely untapped. Investigation of the effect of overexpression of *KN1* genes in monocots on the lignin biosynthesis pathway and the potential effects on downstream plant growth parameters is important. Therefore, the objective of this study was to identify and characterize the switchgrass class I *KNOX* gene (*PvKN1*) and investigate its association with various plant morphological and developmental processes as well as impacts on cell wall chemistry and recalcitrance in switchgrass.

## Materials and Methods

### Plants and Growth Conditions

Transgenic and non-transgenic control switchgrass plants were grown under the same conditions (16 h day/8 h night light at 24°C, 390 μE m^−2^ s^−1^) in growth chambers and watered three times per week, including weekly nutrient supplements with 100 mg/L Peter’s 20-20-20 fertilizer. For quantification of growth parameters, each transgenic and non-transgenic line was propagated from a single tiller to yield three clonal replicates each (Hardin et al., 2013). Leaf blades, leaf sheath, internode sections, and panicle samples pooled from the top two internodes and young roots, were collected from tillers at the R1 developmental stage (Moore et al., 1991; Shen et al., 2009) to assay transcript abundance. Each sample was snap-frozen in liquid nitrogen and pulverized with mortar and pestle in liquid nitrogen. The pulverized samples were used for RNA extraction as described below.

### Gene Identification, Vector Construction, and Transgenic Plant Production

TBLASTN was used to identify the homologous *KNOX* gene sequences from switchgrass EST databases (Zhang et al., 2013) and draft genome (*Panicum virgatum* v1.1, DOE Joint Genome Institute [DOE-JGI], 2015) at Phytozome using the amino acid sequences of ZmKN1 (gb/AAP21616.1) and KNAT1 (At4g08150) as heterologous probes. Subsequently, the most closely related genes (*PvKN1a* and *PvKN1b*) were identified for cloning based on the cluster analysis and multiple sequence alignment analysis. Overexpression cassettes were constructed by isolating target gene ORFs from switchgrass cDNAs of the ST1 clonal genotype of ‘Alamo’ switchgrass using individual gene-specific primers flanking the ORF of each gene and subsequently cloning each into pCR8 entry vector for sequence confirmation. The list of primer pairs used for cloning is shown in Supplementary Table S1. Sequence-confirmed ORFs were then sub-cloned into pANIC-10A expression vector by GATEWAY recombination (Mann et al., 2012) to place each gene of interest under the control of the *ZmUbi1* promoter. Embryogenic callus derived from Alamo switchgrass ST1 or SA37 genotypes (King et al., 2014) was transformed with the expression vector construct using *Agrobacterium*-mediated transformation (Burris et al., 2009). Selection for positive transformants was carried out for approximately 2 months on 30–50 mg/L hygromycin followed by regeneration of OFP reporter-positive callus sections on regeneration medium (Li and Qu, 2011) containing 400 mg/L timentin. Regenerated plants were rooted on MS medium (Murashige and Skoog, 1962) containing 250 mg/L cefotaxime (Grewal et al., 2006) and the transgenic lines were screened based on the presence of the insert and expression of the transgene. The non-transgenic control ST1 and SA37 lines were generated in tissue culture in parallel with the transgenic lines. Rice transformation was performed using callus derived from mature seeds of rice variety TP309 as described before (Nishimura et al., 2006).

### RNA Extraction and qRT-PCR

RNA extraction and analysis of transgene transcripts were performed as previously described (Wuddineh et al., 2015). Briefly, total RNA was extracted from the shoot-tips of transgenic and non-transgenic lines at E4 developmental stage using Tri-Reagent (Molecular Research Center, Cincinnati, OH, USA). Each 3 μg of the purified RNA sample was treated with DNase-I (Promega, Madison, WI, USA) to eliminate any potential genomic DNA contaminants. High-Capacity cDNA Reverse Transcription kit (Applied Biosystems, Foster City, CA, USA) was used for first-strand cDNA synthesis using the DNase-treated RNA. qRT-PCR analysis was conducted using Power SYBR Green PCR master mix (Applied Biosystems) according to the manufacturer’s protocol. All the experiments were conducted in triplicate. Gene-specific forward primers and an AcV5 tag-spanning reverse primer was used for qRT-PCR of the transgenic lines. The list of all primer pairs used for qRT-PCR is shown in Supplementary Table S1. The relative expression was analyzed using the *C*_T_ method using UBQ (Switchgrass Unitranscript ID: AP13CTG25905) as a reference gene (Shen et al., 2009; Xu et al., 2011). No amplification products were observed with all the primer pairs when using only the RNA samples or water instead of cDNA.

### Phloroglucinol Staining

Qualitative lignin analysis was performed as previously described (Wuddineh et al., 2015). Briefly, leaf samples at R1 developmental stage were collected and cleared in a 2:1 solution of ethanol and glacial acetic acid for 5 days (Bart et al., 2010). The third leaves from the base of the stem were used in this experiment as these were the most uniform-looking in terms of maturity. Subsequently, the cleared leaf sample was immersed in 1% phloroglucinol (in 2:1 ethanol/HCl) overnight for staining. Photographs were taken at 1× magnification using infinity X32 digital camera mounted on Fisher Scientific Stereomaster microscope (Pittsburgh, PA, USA).

### Determination of Lignin Content and Composition by Pyrolysis-Molecular Beam Mass Spectrometry

Quantitative lignin analysis and S/G lignin monomer ratio were determined using tillers collected at R1 developmental stage and air dried for 3 weeks at room temperature, followed by milling to 1 mm (20 mesh) particle size. Lignin content and composition were determined at NREL using high-throughput pyrolysis-molecular beam mass spectrometry (pyMBMS) on extractives- and starch-free samples (Sykes et al., 2009; Baxter et al., 2014).

### Determination of Sugar Release

For enzymatic hydrolysis, tiller samples were collected at R1 developmental stage and air-dried for 3 weeks at room temperature before grinding to 1 mm (20 mesh) particle size. Subsequently, sugar release efficiency was determined using high-throughput sugar release assays on extractives- and starch-free samples (Selig et al., 2010; Studer et al., 2010; Decker et al., 2012). Briefly, the milled samples were prepared in a 96-well plate along with biomass-only controls, sugar standards, enzyme-only, and blank wells. The pretreatment was performed in a steam chamber at 180°C for 40 min. The enzyme cocktails used for hydrolysis comprises Novozymes Cellic CTec2 cellulase (Novozymes North America, Franklinton, NC, USA) at 70 mg protein/g initial biomass and Novo188 β-glucosidase (Novozymes) at 2.5 mg protein/g initial biomass. The mixture was incubated for 72 h at 54°C and glucose and xylose release were determined by colorimetric assays. The total sugar release was defined as the sum of glucose and xylose released. Each sample for sugar release assays were run in triplicate.

### Data Analysis

ANOVA with Fisher’s LSD procedure was used to analyze the differences between treatment means while PROC TTEST procedure was used to examine the statistical difference between the expression of target genes in transgenic vs. non-transgenic lines using SAS version 9.3 (SAS Institute Inc., Cary, NC, USA).

## Results

### KNOX Family of TFs in Switchgrass and the Identification of PvKN1

**FIGURE 1:**
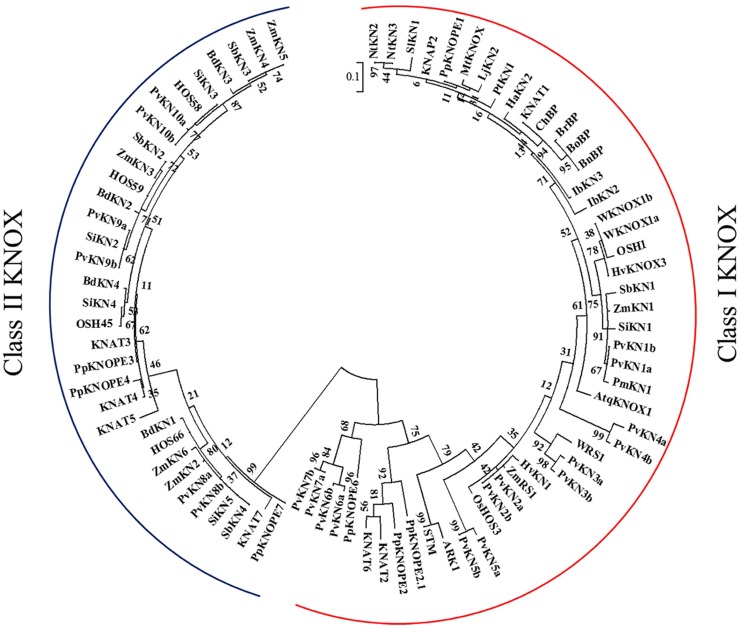
**Maximum likelihood tree of KNOX family of TFs for the deduced amino acid sequences from switchgrass along with already-characterized proteins from both monocots and dicots.** The sequences were aligned using MUSCLE program (Edgar, 2004) and the alignment was curated by Gblocks at the phylogeny.fr (Dereeper et al., 2008). The tree was constructed by maximum likelihood procedure using MEGA6.0 program (Tamura et al., 2013). Analysis using 1000 bootstrap replicates was performed. The scale bar shows 0.1 amino acid substitutions per site. Names of the species and the locus name or GenBank accession numbers of the sequences used in this tree are listed in Supplementary Table S4.

**FIGURE 2:**
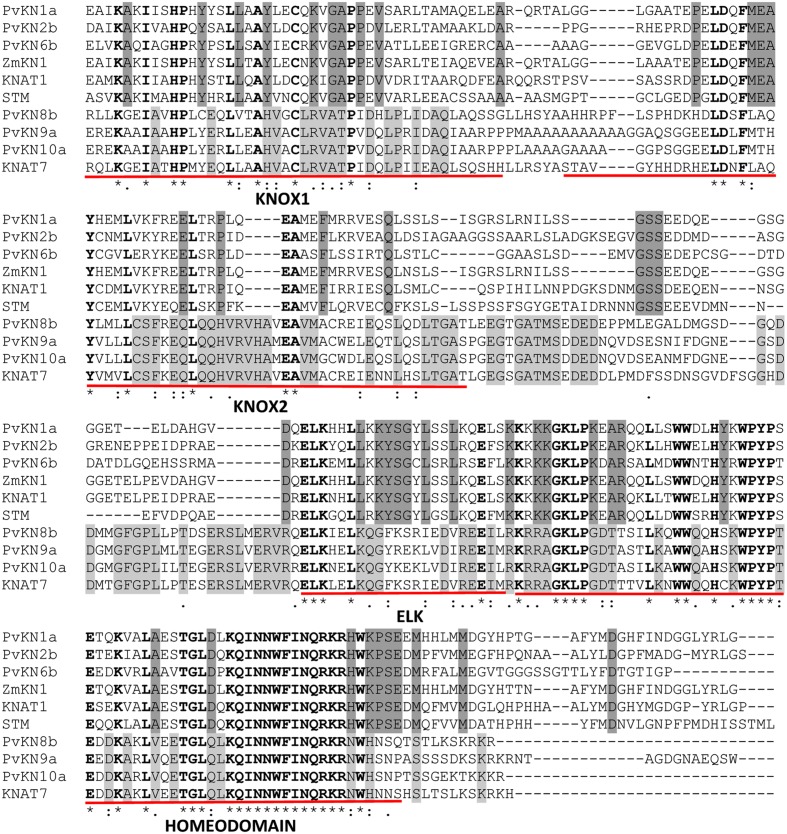
**Multiple amino acid sequence alignment of the C-termini of class I: PvKN1a, PvKN2b, PvKN6b, ZmKN1, KNAT1, and STM and class II (PvKN8b, PvKN9a, PvKN10a, and KNAT7) family of KNOX TFs.** The conserved domains are underlined in red; the strictly conserved amino acid residues are indicated in bold. The amino acid residues specific to class I KNOX TFs are highlighted in dark gray while those specific to class II are highlighted in light gray. The multiple sequence alignment was constructed using the amino acid sequences of respective genes by MUSCLE program (Edgar, 2004).

**FIGURE 3:**
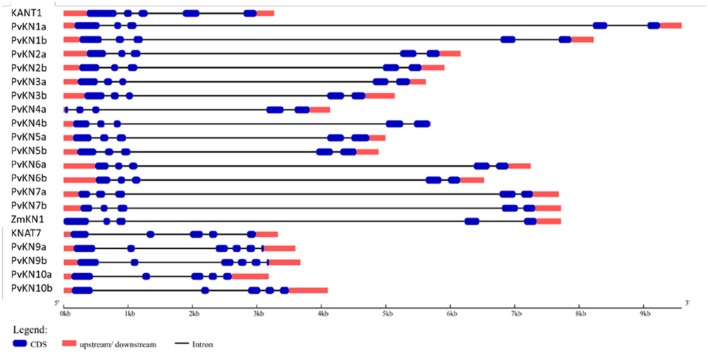
**Gene structures of class I and II KNOX transcription factor family coding genes from *Arabidopsis*, switchgrass (*Panicum virgatum*) and maize (*Zea mays*).** The CDS and the UTR are shown by filled dark blue and pink boxes, respectively. The introns are shown by thick black lines. The gene features were visualized by the gene structure display server (Guo et al., 2007).

A survey of the publicly available switchgrass genome for the characteristic Meinox domain (KNOX1 and KNOX2) and HD revealed a total of 10 genes belonging to the KNOX family of TFs. The tetraploid switchgrass genome of Alamo AP13 (2n = 4x = 36) contains at least two sub-genomic gene variants representing the ‘A’ and ‘B’ subgenomes (**Figure 1**). Phylogenetic and sequence analysis showed seven *KNOX* genes belonging to class I family of KNOX TFs while the remaining three were grouped into the class II family of KNOX TFs (**Figures 1** and **2**). Analysis of the amino acid sequences of the encoded proteins indicated that all share KNOX1, KNOX2, ELK, and HD conserved domains found in the maize KN1, *Arabidopsis* KNAT1 and orthologous TFs in dicots (peach and *Populus*). Moreover, higher identity within members of each class (>44% in class I and >60% in class II) than between the two classes of *KNOX* genes (≤37%) was observed (Supplementary Table S2). Analysis of gene structure among the two classes of *KNOX* genes in comparison to the homologs from *Arabidopsis* (*KNAT1* and *KNAT7*) and maize (*KN1*) showed a highly conserved pattern in each class, typically with five exons and four introns in the genomic DNA with variable length of introns among the genes (**Figure 3**). The exception to this pattern was *PvKN9*, a member of class II *KNOX* genes family, which had an additional intron inserted in the last exon and, therefore, had a structure of six exons and five introns.

Based on amino acid sequence analysis, sub-genomic variants belonging to class I family of KNOX TFs, designated as PvKN1a, PvKN1b, PvKN2a, and PvKN2b, had the highest identity to the maize KN1 and *Arabidopsis* KNAT1 when compared with the other switchgrass class I family members of KNOX TFs (**Figure 2**; Supplementary Table S3). However, based on phylogenetic analysis of the KNOX proteins, PvKN1a and PvKN1b showed more similarity to the group containing the previously characterized KNOX TFs from *Arabidopsis* (KNAT1/BP), maize (ZmKN1), rice (OSH1), wheat (WKNOX1), *Populus* (ARK2), and peach (PpKNOPE1) while PvKN2a and PvKN2b clustered more closely with maize rough sheath 1 (ZmRS1), rice homeobox (OsHOS3) and wheat rough sheath 1 (WRS1) TFs (**Figures 1** and **2**). Moreover, the two gene variants of *PvKN1* (*PvKN1a* and *PvKN1b*) with 95% identity in the predicted amino acid sequences shared over 88% identity with *ZmKN1* as opposed to 54% for *PvKN2a* or *PvKN2b* (Supplementary Table S3). More importantly, comparison of the HD domain, essential for binding target sequences, showed over 98% identity between the PvKN1 and ZmKN1 proteins versus 89% between PvKN2 and ZmKN1 (Supplementary Table S3). Moreover, PvKN1 displayed the highest conservation at the glycine, serine and glutamic acid-rich motif (also known as GSE) (the stretch of amino acids between the KNOX2 and ELK domains) compared with PvKN2 (Supplementary Table S3).

### Expression Patterns of *PvKN1* Variants

**FIGURE 4:**
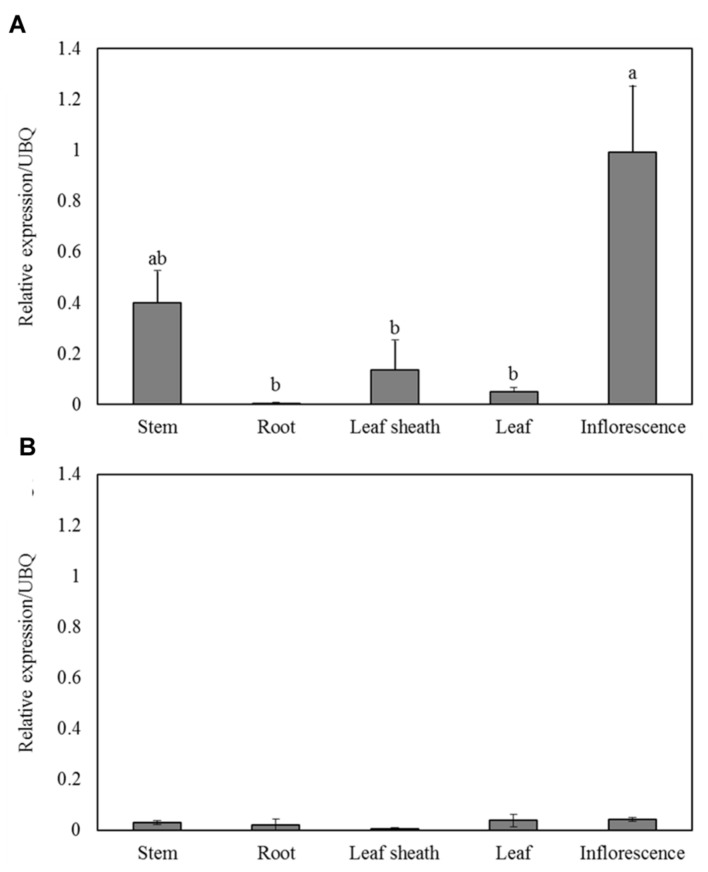
**Expression patterns of *PvKN1a***(A)** and *PvKN1b***(B)** in different plant tissues as determined by qRT-PCR.** Plant samples for RNA extraction used in the qRT-PCR experiments were collected at R1 (reproductive stage 1) developmental stage. The dissociation curve for the qRT-PCR products showed that the primers were gene-specific. The relative levels of transcripts were normalized to UBQ. Bars represent mean values of three replicates ± standard error. Bars represented by different letters are significantly different at *P* ≤ 0.05 as tested by the LSD method with SAS software (SAS Institute Inc.).

Quantitative reverse transcription polymerase chain reaction analysis showed detectable levels of expression for both *PvKN1* gene variants in stems, leaves, leaf sheaths and inflorescences at the R1, the same stage when sugar release, lignin content and growth parameters were analyzed (**Figure 4**). The relative transcript abundance of *PvKN1a* appears to be higher than that of *PvKN1b* in stems, leaf sheath and young inflorescences. Moreover, *PvKN1a* expression was the highest in young inflorescence tissue followed by stems, with the lowest expression observed in roots and leaves (**Figure 4**). The expression of *PvKN1b* was not significantly different among tissues assayed (**Figure 4**).

### Constitutive Overexpression of *PvKN1* in Switchgrass

The switchgrass homologs of maize *KN1* gene, *PvKN1a* and *PvKN1b* were cloned from cDNA and constitutively overexpressed in switchgrass under the control of the ZmUbi1 promoter. In addition, the same *PvKN1a* construct was overexpressed in rice to examine its effect on plant development. Only transgenic calli expressing the OFP marker gene were regenerated (Supplementary Figure S1). Five independent transgenic switchgrass lines of ST1 background overexpressing *PvKN1a* and eight *PvKN1b*-overexpressing lines were recovered.

### Altered Growth Phenotypes in Transgenic Plants Overexpressing *PvKN1*

**FIGURE 5:**
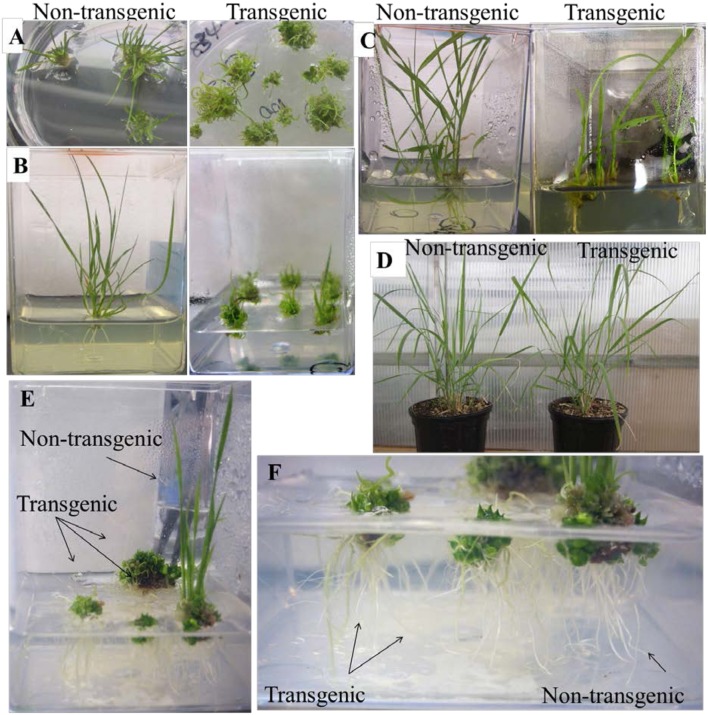
**Altered growth phenotypes in transgenic *PvKN1a*- overexpressing switchgrass (ST1) and rice plants.** **(A)** Transgenic plants overexpressing *PvKN1a* exhibited reduced shoot elongation and curly leaves compared to the normal phenotype in non-transgenic lines. **(B)** Transgenic plants with substantially reduced growth compared to the non-transgenic lines at 5 weeks after incubation on the rooting media. **(C)** Transgenic plants with elongated shoots compared to the non-transgenic lines at 9 weeks after incubation on the rooting media. **(D)** Morphological phenotypes in 3 months old transgenic lines overexpressing *PvKN1a* compared to non-transgenic lines. **(E)** Transgenic rice showing abnormal shoot development compared to the normal phenotype in the non-transgenic plants. **(F)** Transgenic rice showing normal root development as well as the non-transgenic plants.

**FIGURE 6:**
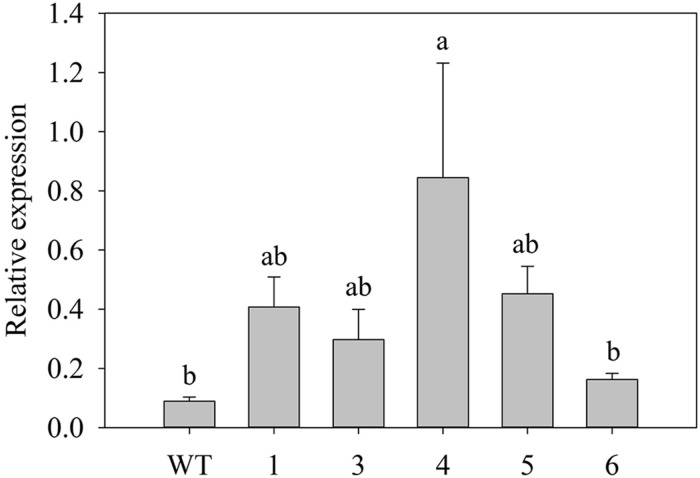
**Relative transcript levels of the *PvKN1a* in transgenic and non-transgenic (WT) switchgrass (ST1) plants.** The expression analysis was done using RNA derived from the shoot tips at E4 developmental stage. The dissociation curve for the qRT-PCR products showed that the primers were gene-specific. The relative levels of transcripts were normalized to UBQ. Bars represent mean values of three replicates ± standard error. Bars represented by different letters are significantly different at *P* ≤ 0.05 as tested by LSD method with SAS software (SAS Institute Inc.).

The transgenic lines overexpressing the two *PvKN1* gene variants had similar phenotypes, which suggests that each variant may have similar functions upon overexpression (Supplementary Figure S2). Therefore, subsequent work focused on detailed analysis of *PvKN1a* overexpressing lines. We observed unusually narrow leaf blades with sheath-like tissues and curled leaves just after plants were regenerated. In addition, the *PvKN1a* overexpression lines had severely inhibited shoot and root elongation as compared with the non-transgenic controls at similar developmental stages (**Figures 5A,B**). Many of the transgenic lines recovered were dwarfed and some were not fully regenerable. However, several transgenic lines that were regenerated were able to develop into mature plants displaying less severe phenotypes after establishment in the rooting media (**Figure 5C**); some of these lines eventually grew normally (**Figure 5D**). The successfully established transgenic lines were confirmed by genomic PCR using transgene and hygromycin-resistance gene specific primers, as well as visualization of OFP in transgenic plants compared with the non-transgenic control lines (Supplementary Figures S1A–S1C). Moreover, there were two to ninefold increases in the *PvKN1* transcript levels in transgenics relative to control lines (**Figure 6**). There were no statistically significant differences in tiller height, tiller number or biomass between transgenic lines and the non-transgenic control (**Table 1**).

**Table 1:** Morphology and biomass yields of transgenic switchgrass lines per plant overexpressing *PvKN1* and non-transgenic control (WT) parent plants.

Lines	Tiller height (cm)	Tiller number	Fresh weight (g)	Dry weight (g)
**1**	50.7 ± 1.6^a^	7.5 ± 0.5^a^	24.8 ± 6.5^a^	7.5 ± 1.2^a^
**3**	40.5 ± 1.7^b^	12.7 ± 4.5^a^	24.3 ± 11.8^a^	9.3 ± 4.3^a^
**4**	45.0 ± 3.4^ab^	10.3 ± 4.2^a^	21.9 ± 3.2^a^	8.4 ± 0.4^a^
**WT**	45.3 ± 2.5^ab^	9.7 ± 1.3^a^	27.7 ± 9.3^a^	10.7 ± 1.3^a^

*Tiller height estimates were determined for each plant by taking the mean of the five tallest tillers within each biological replicate. The fresh and dry biomass measurements were taken on 4-mo-old aboveground plant biomass harvested at similar growth stages (reproductive stage R1). Values are means of three biological replicates ± standard errors (n = 3). Values represented by different letters are significantly different at P ≤ 0.05 as tested by the LSD method with SAS software (SAS Institute Inc.).*

Overexpression of *PvKN1* in a different switchgrass genotype (SA37) background showed abnormal phenotypes at later stages of growth compared to the genotype (ST1) used in this study. These transgenic lines had relatively shorter internodes and twisted or bent culms with altered leaf structure including curly leaves, fused leaf sheath-blade boundary and missing ligules (Supplementary Figures S3A–S3C). The relative expression of the *PvKN1* transcript was >50-fold in transgenic compared to the non-transgenic control lines (Supplementary Figure S3D).

When *PvKN1* was overexpressed in rice, similar aberrant phenotypes were observed with clumps of vegetative shoots developed from the transgenic callus after incubation on the shoot regeneration medium. In general, shoot elongation was severely reduced in rice even after longer incubation on the growth medium while root growth was apparently unaffected (**Figures 5E,F**).

### Overexpression of *PvKN1* in Switchgrass and Subsequent Changes in the Expression of Putative Target Genes in Transgenic Plants

**FIGURE 7:**
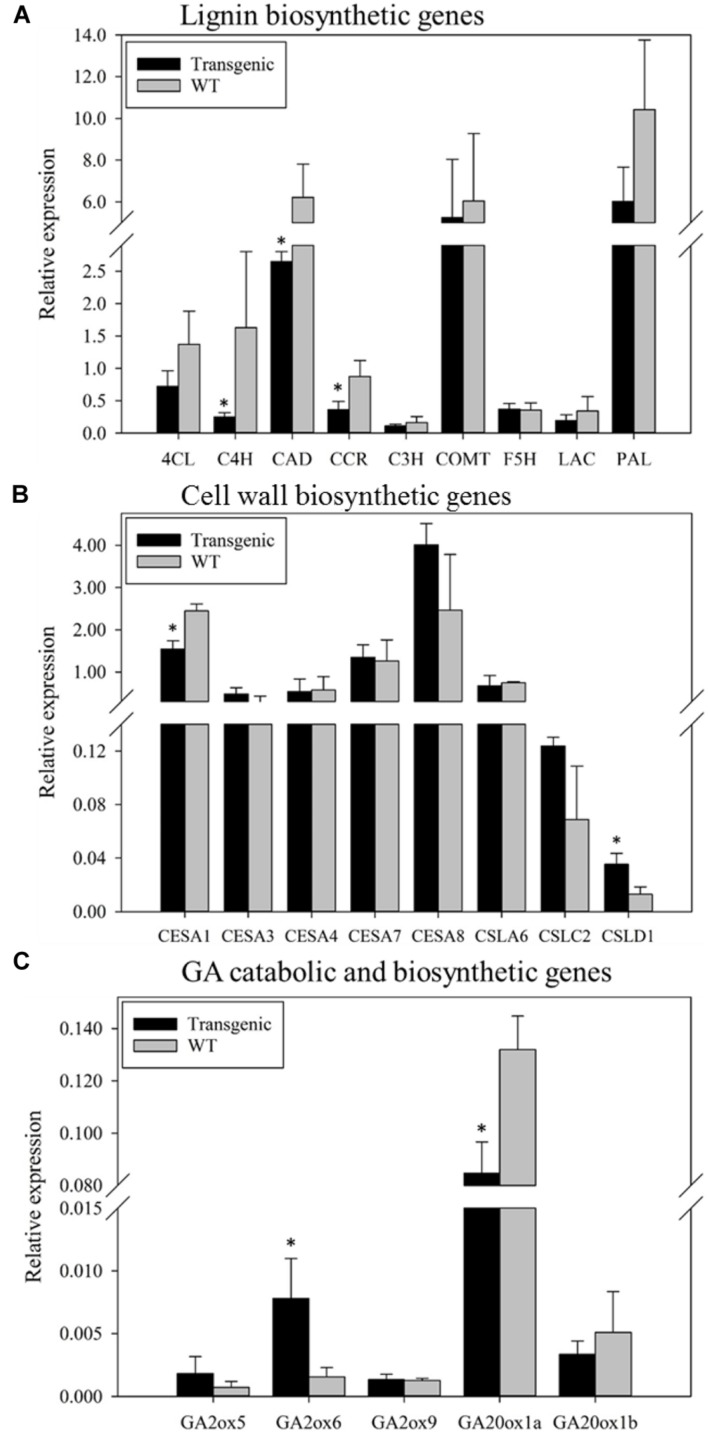
**The relative expression of putative target genes of PvKN1 in transgenic switchgrass (line 4) overexpressing *PvKN1* vs the non-transgenic (WT) plants as determined by qRT-PCR.** **(A)** Lignin biosynthetic genes. **(B)** Cell wall biosynthetic genes. **(C)** Gibberellin (GA) catabolic and biosynthetic genes. The relative levels of transcripts were normalized to UBQ. Asterisks indicate significant differences from non-transgenic control plants at *P* ≤ 0.05 as determined by PROC TTEST procedure using SAS software (SAS Institute Inc.). Bars represent mean values of three replicates ± standard error. The lignin and GA biosynthetic and catabolic genes were as described before in Wuddineh et al. (2015). The cellulose and hemicellulose biosynthetic genes were labeled according to the naming from the closest rice or *Arabidopsis* homologs used in Ambavaram et al. (2011) (Supplementary Figure S5). (Pv)4CL (4-coumarate: CoA ligase); (Pv)C3H (coumaroyl shikimate 3-hydroxylase); (Pv)C4H (coumaroyl shikimate 4-hydroxylase); (Pv)CAD (cinnamyl alcohol dehydrogenase); (Pv)CCR1 (cinnamoyl CoA reductase1); (Pv)COMT (caffeic acid 3-*O*-methyltransferase); (Pv)F5H (ferulate 5-hydroxylase); (Pv)PAL (phenylalanine ammonia-lyase); (Pv)LAC1 (laccase1); (Pv)CESA (cellulose synthase); (Pv)CSL (cellulose synthase-like).

To test whether overexpression of *PvKN1* in switchgrass could affect the expression of putative target genes, qRT-PCR analysis was conducted. *PvKN1*-overexpressing switchgrass displayed significantly reduced expression of lignin biosynthetic genes including *PvC4H*, *PvCAD*, and *PvCCR1* compared with the control (**Figure 7A**). The expression of putative cellulose and hemicellulose biosynthetic genes were also evaluated in transgenic and non-transgenic lines. The results showed that the relative transcript level of putative primary cell wall cellulose biosynthetic gene *PvCESA1* was significantly reduced, while the expression of other putative primary cell wall biosynthetic gene, *PvCESA3* and that of three secondary cell wall associated putative *PvCESA* genes *PvCESA4*, *PvCESA7*, and *PvCESA8*, were unchanged in transgenic compared with the control lines. A significant increase in the expression level of the putative hemicellulose biosynthetic gene, *PvCSLD1* was also observed in transgenic compared to the control lines (**Figure 7B**). The expression of putative GA signaling pathway genes were also evaluated in transgenic and non-transgenic lines. The results showed that the expression of putative *PvGA20ox* gene (*PvGA20ox1a*) was significantly reduced in transgenic compared to the non-transgenic control lines. In contrast, the relative expression level of one (*PvGA2ox6*) of the three C_20_ *GA2ox* genes (responsible for catabolism of the 20-carbon precursors of bioactive GA) evaluated in this study showed a significant sixfold increase in transgenic switchgrass compared with non-transgenic controls (**Figure 7C**).

### Changes in Lignin Content and Composition in Transgenic Switchgrass

**FIGURE 8:**
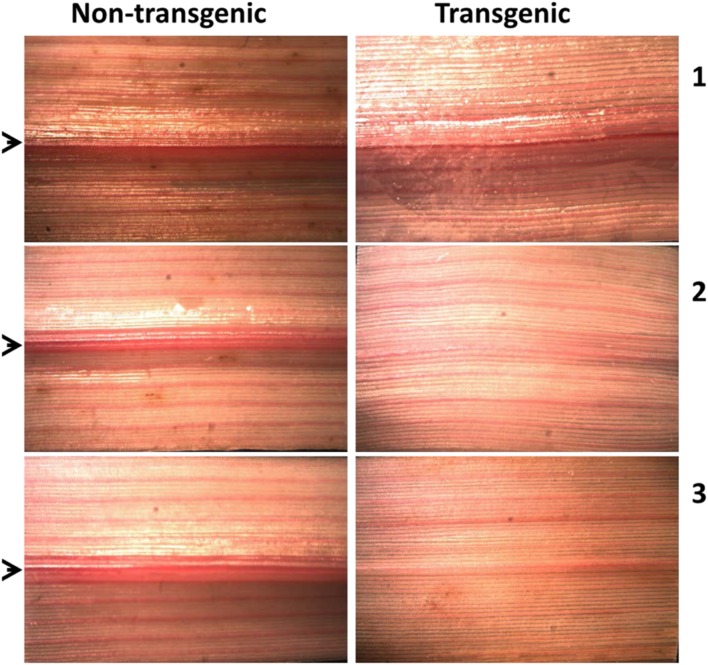
**Histochemical detection of lignin in leaves of *PvKN1a* overexpressing and non-transgenic control switchgrass lines using light microscopy.** The phloroglucinol-HCl staining was done on leaves from three independent tillers indicated by numbers **(1–3)**. The images were taken at 1× magnification. The arrows indicate midribs of leaves.

There appeared to be reduced lignin in transgenic leaves stained with phloroglucinol-HCl (**Figure 8**). Moreover, quantitative analysis of total lignin content in whole tillers by pyMBMS from the R1 developmental stage showed a slight reduction in lignin content by up to 8% in the transgenic lines relative to controls (Supplementary Figure S4A). The S/G lignin monomer ratio in R1 tillers of transgenic lines was not different from that in the non-transgenic control (Supplementary Figure S4B).

### Effect of Overexpression of *PvKN1* on Sugar Release Efficiency in Switchgrass

**FIGURE 9:**
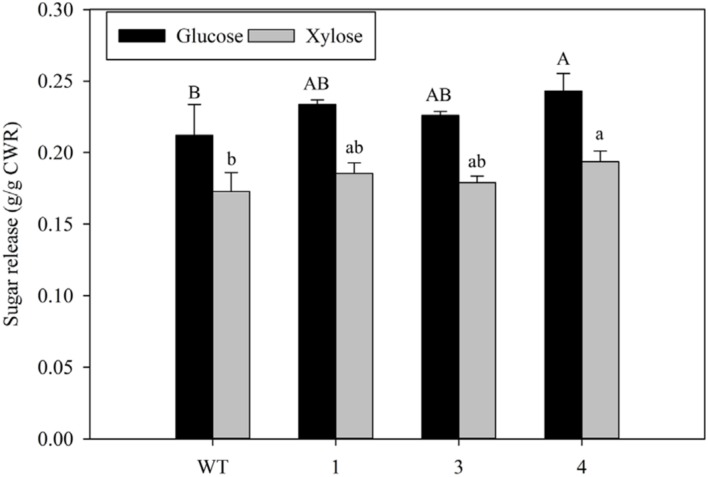
**Sugar release by enzymatic hydrolysis in transgenic and non-transgenic control (WT) switchgrass lines.** All data are means ± standard deviation (*n* = 3). Bars represented by different letters are significantly different at *P* ≤ 0.05 as tested by the LSD method with SAS software (SAS Institute Inc.). CWR, cell wall residues.

Sugar release in R1 whole tillers was significantly increased in transgenic line 4, which had the highest transgene expression among lines, in which 15% more glucose and 12% more xylose was released as compared with the non-transgenic control. The total sugar release (glucose and xylose combined) in this line was increased by up to 13% relative to the control (**Figure 9**).

## Discussion

### Structural Conservation in Switchgrass *KNOX* Genes and Functional Implications

In this study, we identified 10 putative KNOX TF genes in the switchgrass genome, which include seven members of the class I and three of the class II families (**Figure 1**; Supplementary Table S3). So far, a total of 13 *KNOX* genes in maize, 12 in rice, 15 in *Populus*, 8 in *Arabidopsis*, 10 in peach, 5 in *Selaginella moellendorffii*, 5 in moss and 1 in *Chlamydomonas reinhardtii* have been reported (Mukherjee et al., 2009; Hay and Tsiantis, 2010; Testone et al., 2012). Thus, it appears that tetraploid switchgrass could harbor more *KNOX* genes than we have reported here, which may be revealed as the more complete genome sequences are made available. In general, the identified switchgrass *KNOX* genes showed high similarity to homologs in other plant species in terms of gene structure, amino acid sequence identity of deduced proteins, with shared specific domains (**Figures 1–3**, Supplementary Table S3). All members share the characteristic domains found in the maize KN1 including Meinox (essential for suppressing target gene expression and homodimerization), ELK (nuclear localization signal) and HD (**Figure 2**) (Kerstetter et al., 1994; Nagasaki et al., 2001). Specifically, PvKN1 from class I family KNOX TFs had the highest amino acid sequence identity (>90%) with the well-characterized maize KN1 but even more identity (>98%) in the HD domain (**Figure 2**, Supplementary Table S3). These remarkable structural resemblances between the *KNOX* genes among both monocots and dicots perhaps reflect functional conservation among homologs, which is supported by functional studies (Mele et al., 2003; Du et al., 2009; Testone et al., 2012; Townsley et al., 2013).

### *PvKN1* Gene Expression is Mainly Associated with Meristem Development

Most class I *KNOX* genes are highly expressed in meristematic tissue in accordance with their proposed function in the maintenance of meristem identity (Kerstetter et al., 1994, 1997; Chuck et al., 1996; Tioni et al., 2003; Testone et al., 2008). Similarly, *PvKN1* was also expressed mainly in stem and inflorescence tissues containing meristems, in line with the importance of KNOX homologs in the regulation of apical or intercalary meristem development in young tissues of other species (Kerstetter et al., 1994, 1997). It is interesting to note that *PvKN1* transcripts, albeit at low levels, were detected in differentiated lateral organs, including leaves and leaf sheaths, in contrast to the situation in maize, rice, *Arabidopsis* and *Populus* where expression of *KNOX* genes is scantly detected in such tissues (Matsuoka et al., 1993; Kerstetter et al., 1994; Chuck et al., 1996; Du et al., 2009). However, similar expression pattern of *KNOX* genes in differentiated tissues has been reported in barley, sunflower and tomato (Hareven et al., 1996; Muller et al., 2001; Tioni et al., 2003). *KNOX* gene expression in tomato leaves was associated with compound leaf development (Hareven et al., 1996). One possible reason for the lack of *KNOX* gene expression phenotype (ectopic SAM formation) in differentiated switchgrass tissues might be the low transcript level, which might be below the required threshold level to induce the phenotype. Moreover, the involvement of various cell and species-specific factors such as posttranscriptional regulators in the determination of developmental responses to the expression of *KN1-*like genes might also contribute to this deviation as previously suggested (Tioni et al., 2003; Hay and Tsiantis, 2010). Whether or not PvKN1 has a role in non-meristematic tissues remains to be seen.

### Overexpression of *PvKN1* Differentially Regulate Plant Growth Phenotypes in Switchgrass and Rice

Ectopic expression of *KNOX* genes have been shown to modify plant growth and development in both monocots and dicots (Matsuoka et al., 1993; Sentoku et al., 2000; Du et al., 2009; Hay and Tsiantis, 2010). Similarly, ectopic overexpression of *PvKN1* in switchgrass dramatically altered growth phenotype, including plant architecture, leaf structure, and shoot elongation in transgenic plants especially, at early developmental stages (**Figure 5B**; Supplementary Figure S3B). There was a gradation of phenotype severity in different transgenic lines. In the worst-case, growth was arrested at the early seedling stage (**Figure 5A**), whereas other lines eventually attained normal shoots and roots after prolonged incubation on the rooting medium (**Figure 5C**) even though *PvKN1* transcript level in these lines was up to nine times higher than the control. In contrast, higher levels of *PvKN1* transcripts (upward of 50-fold increase) in transgenic lines in the SA37 switchgrass genotype background displayed aberrant phenotypes even at later stages of development (Supplementary Figure S3). The apparent phenotypic differences in these transgenic plants might be attributed to the differences in transcript abundance as well as the genotypic differences in response to *PvKN1* overexpression as previously reported for maize (Kerstetter et al., 1997; Foster et al., 1999). The dependence of phenotype severity on *KNOX* transcript level has been reported in rice and *Arabidopsis* (Sentoku et al., 2000; Hay et al., 2003).

Interestingly, ectopic expression of *PvKN1* failed to induce ectopic meristem development in lateral organs, in contrast to other plants with simple leaves such as maize *KN1* dominant gain-of-function mutants (Vollbrecht et al., 1991), rice overexpressing *OSH1* (Matsuoka et al., 1993) or *Populus* overexpressing *ARK1* (Groover et al., 2006). However, differences in the effect of *KNOX* gene overexpression may be related to levels or patterns of ectopic overexpression. Consistent with this idea, no aberrant meristems or shoot phenotypes were observed in dicots such as tobacco that overexpressed the rice *OSH1* gene (Kano-Murakami et al., 1993), and *Populus* that overexpressed *ARK2* genes (Du et al., 2009), or in rice overexpressing *OSH1* under a weak CaMV 35S promoter (Sentoku et al., 2000). On the other hand, *PvKN1* overexpressing plants clearly exhibited altered distal-proximal patterns of leaf growth where the development of distal leaf blades was inhibited, which, in turn, gave rise to the development of proximal leaf sheath-like tissues as previously reported in rice and maize expressing *KNOX* genes (**Figure 5A**; Supplementary Figure S3A; Vollbrecht et al., 1991; Sentoku et al., 2000). This observation is in accordance with the maturation schedule hypothesis, which proposes that leaf primordia progress through a series of developmental stages along the proximal to distal axis of the leaf to form distinct tissue types, namely sheath, ligule, auricle and leaf blade, and ectopic expression of *KNOX* genes causes developmental delays impeding cells from normal progression through subsequent phases of maturation schedule (Freeling, 1992).

Another intriguing observation was the twisted stem and altered leaf phenotypes in SA37 switchgrass plants overexpressing *PvKN1*, which resembled the phenotypes observed in dominant mutants of maize *KNAT4* (*Gnarley1*; Foster et al., 1999). This specific phenotype was not observed in rice and maize overexpressing the homologs of PvKN1 (Vollbrecht et al., 1991; Sentoku et al., 2000). The diverse effects of ectopic *KNOX* gene expression in lateral organs may reflect the differential competence of tissues in addition to the transcript level in dictating tissue response to *KNOX* gene action. Two major factors have been suggested to modulate the competence of leaf tissues in *Arabidopsis* and tomato, i.e., KNOX-independent regulation of common target genes, and the regulation of tissue differentiation (Hay and Tsiantis, 2010). The interaction between different KNOX proteins and BELL or OVATE proteins might, in turn, be involved in the regulation of these factors (Bolduc et al., 2014). In general, such differences in the phenotypes among homologous gene expression could also indicate divergence in the gene regulatory mechanisms of KNOX genes among plant species as radial adaptation.

Rice with *PvKN1* overexpression produced clumps of multiple shoots, but with no aberrant effect on root development. These phenotypes were consistent with the previous findings in rice where class I *KNOX* genes such as *OSH1* and *OSH3* were overexpressed (Sentoku et al., 2000; Nagasaki et al., 2001), further supporting the homology between these genes. The difference between switchgrass and rice in response to the expression of *PvKN1* might be attributed to differential *cis*-regulation of the gene by different regulatory elements, as suggested by Hay and Tsiantis (2010). Taken together, these results hinted that the mechanism behind PvKN1-induced regulation of genes and downstream phenotypes is complex and may differ from species to species.

### *PvKN1* Expression is Associated with the Regulation of Cell Wall Biosynthesis via Regulation of GA and BR Signaling Pathways

*PvKN1* overexpression resulted in reduced lignin deposition in the plant biomass accompanied by reduced expression of lignin biosynthetic genes including *PvC4H*, *PvCAD*, and *PvCCR1* (**Figure 7A**). This observation is generally in line with the previous reports in *Arabidopsis*, peach and *Populus* (Mele et al., 2003; Du et al., 2009; Testone et al., 2012). Interestingly, *PvKN1* overexpression appeared to downregulate the expression of a primary cell wall cellulose biosynthetic gene, *PvCESA1*, while upregulated the expression of hemicellulose biosynthetic gene (*PvCSLD1*; **Figure 7B**) indicating that PvKN1 may be involved in the regulation of cell wall biosynthetic genes. Unlike overexpression of *ARK2* in *Populus*, which downregulated the expression of three secondary cell wall cellulose biosynthetic genes (Du et al., 2009), no significant alteration of expression of these genes was observed in *PvKN1*-overexpressing transgenic switchgrass (**Figure 7B**). This difference might be attributed to the biological differences between the two species or the differences between the tissues analyzed. However, the regulatory mechanism of cell wall biosynthesis involving KN1 TFs is less clear.

It was recently suggested that the maize *KN1* gene regulates the expression of genes responsible for the biosynthesis of lignin, and perhaps other cell wall components indirectly via the regulation of ‘executive’ genes (Bolduc et al., 2012). However, the direct regulation of a few lignin biosynthetic genes including *COMT* and *CCoAOMT* in *Arabidopsis* (Mele et al., 2003), and *CCoAOMT* in peach (Testone et al., 2012) has also been demonstrated. The GA-signaling pathway has been reported as one of the direct targets of KN1 through which other plant morphological and biochemical characteristics are regulated (Bolduc et al., 2012). In this study, we showed that overexpression of *PvKN1* downregulated the expression of putative *PvGA20ox* (the rate-limiting enzyme in the GA biosynthesis pathway) while upregulated the expression of C_20_ *PvGA2ox* genes (**Figure 7C**) suggesting that PvKN1 might limit shoot elongation and lignification at early growth stages via regulation of GA biosynthetic and catabolic genes and GA signaling.

The mechanism whereby PvKN1 regulates cellulose biosynthetic genes (**Figure 7B**) is unknown. One possible scenario is that PvKN1 regulates the expression of cellulose biosynthetic genes through modulation of BR signaling, which has been shown to positively regulate the expression of cellulose biosynthetic genes (Xie et al., 2011). This hypothesis is supported by the recent report that maize KN1 could interact with BR biosynthetic and catabolic genes (Bolduc et al., 2012), and OSH1 regulates BR-signaling in rice (Tsuda et al., 2014).

### Implications of PvKN1 TF for Improvement of Bioenergy Crops

The prospect of drastically decreasing lignin by ectopic expression of *KNOX* genes motivated our study in switchgrass. The challenge here, as with the case of transgenic switchgrass in which *MYB4* (Shen et al., 2012) and *miRNA156* (Fu et al., 2012) gene expression was changed, is to empirically determine the optimal TF transgene expression level that decreases lignin and possibly provides favorable plant architecture changes, without reducing shoot growth. Clearly, if *KN1* expression is too high, plants are dwarfed and abnormal. Indeed, relatively low *PvKN1* overexpression resulted in reduced lignin deposition/biomass recalcitrance and improved sugar release efficiency without significantly affecting various growth parameters such as tiller height, tiller number, fresh and dry biomass weight (**Figure 8** and **9**; **Table 1**). Therefore, the observed increase in sugar release in transgenic switchgrass with reduced lignin content highlight the potential biotechnological application of *PvKN1* for the improvement of biomass characteristics for bioenergy feedstocks as well as forage grasses. Previous studies have suggested that expression of *KNOX* genes may enhance cytokinin levels (Frugis et al., 2001; Jasinski et al., 2005; Yanai et al., 2005) while cytokinin accumulation was shown to increase drought tolerance via coordinated regulation of carbon and nitrogen assimilation (Peleg et al., 2011; Reguera et al., 2013). Whether *PvKN1* expression plays similar roles should be the subject of future investigation as such traits may enhance the value of using transgenic lines as improved bioenergy feedstocks.

## Conclusion

In summary, we identified a gene coding for class I KNOX TF in switchgrass, *PvKN1*, as a putative ortholog of maize *KN1*. Our results demonstrated that PvKN1 may facilitate shoot elongation and lignification via transcriptional regulation of the GA-biosynthesis pathway. Moreover, we showed that PvKN1 may also be involved in the regulation of the biosynthetic genes of other cell wall polymers (cellulose and hemicellulose) and hence play an important role in cell wall biosynthesis. *PvKN1*-overexpressing lines displaying normal growth phenotypes but with reduced recalcitrance to enzymatic saccharification could potentially be utilized for the improvement of lignocellulosic bioenergy feedstocks for biofuels or for the improvement of forage grass. *PvKN1* could also be potentially used in gene-stacking studies along with genes imparting novel traits, such as higher biomass yield, biotic or abiotic stress resistance to develop more improved bioenergy feedstock.

## Author Contributions

WW designed and performed the experiments, analyzed the data, and drafted the manuscript. MM participated in experimental design and data analysis, assisted with revisions to the manuscript and coordination of the study. J-YZ and MU assisted with cloning of the target gene and contributed in revision of the manuscript. GT, RS, SD, and MD assisted with performing lignin and sugar release assays and contributed in revision of the manuscript. CNS conceived the study and its design and coordination, and assisted with revisions to the manuscript. All authors read and consented to the final version of the manuscript.

## Conflict of Interest Statement

The authors declare that the research was conducted in the absence of any commercial or financial relationships that could be construed as a potential conflict of interest.

## Abbreviations

ANOVA: analysis of variance
BELL: BEL1-like homeodomain
BR: brassinosteroid
CAD: cinnamyl alcohol dehydrogenase
CDS: coding DNA sequence
CCoAOMT: caffeoyl CoA 3-*O*-methyltransferase
CCR: cinnamoyl CoA reductase
CESA: cellulose synthase
COMT: caffeic acid 3-*O*-methyltransferase
CSL: cellulose synthase-like
CWR: cell wall residues
C3H: coumaroyl shikimate 3-hydroxylase
C4H: coumaroyl shikimate 4-hydroxylase
F5H: ferulate 5-hydroxylase
GA2ox: Gibberellin 2-oxidase
GA20ox: Gibberellin 20-oxidase
HD: homeodomain
KN1: Knotted1
KNAT1: KN1-like from *Arabidopsis*
KNOX: KN1-like homeobox
LAC: laccase
LSD: least significant difference
NAC NAM: ATAF1/2 and CUC2
OFP: orange fluorescent protein
PAL: phenylalanine ammonia-lyase
qRT-PCR: quantitative reverse transcription polymerase chain reaction
R1: reproductive stage 1
SAM: shoot apical meristems
S/G: syringyl/guaiacyl
STM: SHOOT MERISTEMLESS
SWN: secondary wall NAC
TALE: three amino acid loop extension
TF: transcription factor
UBQ: ubiquitin
UTR: untranslated regions
ZmUbi1: maize ubiquitin 1
4CL: 4-coumarate: CoA ligase.
