# Supplementary material for: Identification and Overexpression of a Knotted1-Like Transcription Factor in Switchgrass (Panicum virgatum L.) for Lignocellulosic Feedstock Improvement
Source: Front Plant Sci. 2016 Apr 28;7:520. doi: 10.3389/fpls.2016.00520 (PMC4848298; doi:10.3389/fpls.2016.00520)
Supplement: Supplementary file 1 [file Data_Sheet_1.DOCX]

**Supplementary Table 1** List of primers used in this study.

| **Primer name** | **Primer sequence** |  |
| --- | --- | --- |
| **Primers for Cloning PvKN1a cDNA** | | |
| KNF1 | 5′-ATGGAGGAGATCACCCACC-3′ |  |
| KNR12 | 5′-GCCGAGCCGGTACAGCC-3′ |  |
| **Primers for Cloning PvKN1b cDNA** | | |
| 834 OE-F1 | 5′-ATGGAGGAGATCACCCACCAGTA-3′ |  |
| 834 OE-R1 | 5′-ATAGCCGAGCCGGTACAGCCCGC-3′ |  |
| **Primers for genomic DNA PCR** | | |
| Hygro_F | 5′-TTGCATCTCCCGCCGTTCACAG-3′ |  |
| Hygro_R | 5′-CTGGGGCGTCGGTTTCCACTAT-3′ |  |
| 834_F | 5′-TGGGATCTGCACTACAAATGGCCT-3′ |  |
| 834_R/common | 5′-ACAGCGACTTCCTGACCATCCT-3′ |  |
| **Gene-specific primers for qRT-PCR** | | |
| **Primers for confirming the overexpression of *PvKN1*** | | |
| F_834III | 5′-GGACGGGCACTTCATCAACG-3′ |  |
| R_AcV5 | 5′-ACCAGCCGCTCGCATCTTTC-3′ |  |
| **Gene specific primers for *PvKN1a*:** | | |
| PvKN1_F2 | 5′-ATCTGAGGAAATGCACCACCTG-3′ |  |
| PvKN1_R2 | 5′-ATGAAGTGCCCGTCCATGTAGA-3′ |  |
| **Gene specific primers for *PvKN1b*** | | |
| AP13CTG11002F2 | 5′-CCTGAAATTGATGCACATGGTGTTG-3′ |  |
| AP13CTG11002R2 | 5′-AGTGCAGATCCCACCAGCTAAG-3′ |  |

**Supplementary Table 1** Continued.

| **Primer name** | **Unitranscript ID** | **Primer sequence** | **Reference** |
| --- | --- | --- | --- |
| **Primers for reference gene** | | | |
| F_PvUBIQUITIN | AP13CTG25905 | 5′-CAGCGAGGGCTCAATAATTCCA-3′ | Xu et al., 2011 |
| R_PvUBIQUITIN |  | 5′-TCTGGCGGACTACAATATCCA-3′ | Xu et al., 2011 |
| **Primers for analyzing the expression of lignin biosynthetic genes** | | | |
| C4H1_1534F | AP13CTG28733 | 5′-GGGCAGTTCAGCAACCAGAT-3′ | Shen et al., 2012 |
| C4H1_1611R |  | 5′-CGCGTTTCCGGGACTCTAG-3′ | Shen et al., 2012 |
| PvCOMT_F461 | KanlCTG02872 | 5′-CAACCGCGTGTTCAACGA-3′ | Shen et al., 2012 |
| PvCOMT_R534 |  | 5′-CGGTGTAGAACTCGAGCAGCTT-3′ | Shen et al., 2012 |
| 4CL1_1179_F | AP13CTG06049 | 5′-CGAGCAGATCATGAAAGGTTACC-3′ | Shen et al., 2012 |
| 4CL1_1251_R |  | 5′-CAGCCAGCCGTCCTTGTC-3′ | Shen et al., 2012 |
| PvCCR1. 112_F | AP13ISTG52570 | 5′-GCGTCGTGGCTCGTCAA-3′ | Shen et al., 2012 |
| PvCCR1. 187_R |  | 5′-TCGGGTCATCTGGGTTCCT-3′ | Shen et al., 2012 |
| PvCAD_F116 | KanlCTG19538 | 5′-TCACATCAAGCATCCACCATCT-3′ | Shen et al., 2012 |
| PvCAD_R184 |  | 5′-GTTCTCGTGTCCGAGGTGTGT-3′ | Shen et al., 2012 |
| PAL_F1 | KanlCTG00004 | 5′-CATATAGTGTGCGTGCGTGTGT-3′ | Wuddineh et al., 2015 |
| PAL_R1 |  | 5′-CTGGCCCGCCAATCG-3′ |  |
| C3H_F1 | AP13ISTG41630 | 5′-CGTGAACAATGGGATCAGGATAG-3′ | Wuddineh et al., 2015 |
| C3H_R1 |  | 5′-GCGGACACAACCATCTCAAATAC-3′ |  |
| F5H_F1 | AP13ISTG56842 | 5′-CCCCGTGCACTGACGATCTAT-3′ | Wuddineh et al., 2015 |
| F5H_R1 |  | 5′-CCAAGCCAAGGGAAAACACAGTTA-3′ |  |
| Laccase_F2 | AP13CTG11594 | 5′-AGCATCCTGGGCATCGAGAG-3′ | Figure S5A |
| Laccase_R2 |  | 5′-GTCGTAGTTGCCGTACCCTTG-3′ |  |
| **Primers for cellulose and hemicellulose synthetic genes** | | | |
| CESA1_F1 | AP13CTG06092 | 5′-GCATCCAGGGTCCAGTTTATGTG-3′ | Figure S5C |
| CESA1_R1 |  | 5′-CCAGATCGGCTTCGGTCAATAC-3′ |  |
| CESA3_F1 | AP13CTG00607 | 5′-GCATTTCCCTCCTCGTCGTC-3′ | Figure S5C |
| CESA3_R1 |  | 5′-ACTTGCTCCTCTCGCTCTAGC-3′ |  |

**Supplementary Table 1** Continued.

| **Primer name** | **Unitranscript ID** | **Primer sequence** | **Reference** |
| --- | --- | --- | --- |
| CESA4_F1 | AP13CTG01684 | 5′-GAATGCTCTGGTCCGAGTGTC-3′ | Figure S5C |
| CESA4_R1 |  | 5′-TCTGCCAACAGTAGGGTCCATC-3′ |  |
| CESA7_F1 | AP13CTG19403 | 5′-CTTCTCGCTCGTCTGGGTTAG-3′ | Figure S5C |
| CESA7_R1 |  | 5′-AGCTCGATCAATTCAGCACTCG-3′ |  |
| CESA8_F1 | AP13CTG00048_1 | 5′-CGTGAGAAGCGTCCTGGATTC-3′ | Figure S5C |
| CESA8_R1 |  | 5′-CCTGAGAGCCTTGCTGTTGTTG-3′ |  |
| CSLA6_F1 | AP13CTG14018 | 5′-TGCACATTACGGAGCTTGGTG-3′ | Figure S5C |
| CSLA6_R1 |  | 5′-TGGTCCCTCCCATACGCAAG-3′ |  |
| CSLC2_F1 | AP13CTG06284 | 5′-GTAGAAGCAGCCAAGGCACTG-3′ | Figure S5C |
| CSLC2_R1 |  | 5′-GAGTCGCTGTACGCCTCTTG-3′ |  |
| CSLD1_F1 | AP13CTG08033 | 5′-CACAGCTACCACGTCCACATC-3′ | Figure S5C |
| CSLD1_R1 |  | 5′-CGATGACCTTGTCCATGAGGTG-3′ |  |
| **Primers for gibberellin biosynthetic and catabolic genes** | | | |
| 46534F1 | Pavirv00046534 | 5′-ACACCGACAGCGACTTCCTC-3′ | Wuddineh et al., 2015 |
| 46534R1 |  | 5′-GGTCTCCGACGTTGACGATG-3′ |  |
| 35270F2 | Pavirv00035270m | 5′-CAAGAGCGTGGAGCACAAGG-3′ | Wuddineh et al., 2015 |
| 35270R2 |  | 5′-CGAAGGTGAAGGTCCTGTAGG-3′ |  |
| KanlCTG23388F1 | KanlCTG23388 | 5′-AGCGTGGAGCACAAGGTGATG-3′ | Wuddineh et al., 2015 |
| KanlCTG23388R1 |  | 5′-TCTTCCTGCACCTTCCGTCTG-3′ |  |
| GA20-oxF1 | AP13ISTG69826 | 5′-CTGCTGCTCTGATCTGCTCCTG-3′ | Figure S5B |
| GA20-oxR1 |  | 5′-GCAGGCAGGCATACATCGTCT-3′ |  |
| GA20-oxF3 | AP13ISTG41447 | 5′-GCTCTCGCTGGAGATCATGGAG-3′ | Figure S5B |
| GA20-oxR3 |  | 5′-GGAGTCGTTCCCCTCGAAGAAG-3′ |  |

**Supplementary Table 2** Percent identity matrix of the switchgrass KNOX transcription factors.

PvKN1a PvKN1b PvKN2a PvKN2b PvKN3a PvKN3b PvKN4a PvKN4b PvKN5a PvKN5b PvKN6a PvKN6b PvKN7a PvKN7b PvKN8a PvKN8b PvKN9a PvKN9b PvKN10a PvKN10b

PvKN1a

PvKN1b 96.24

PvKN2a 57.01 58.10

PvKN2b 56.48 57.86 96.44

PvKN3a 49.85 51.40 72.67 72.29

PvKN3b 49.85 51.55 70.93 71.20 93.73

PvKN4a 54.81 54.13 59.59 59.59 58.02 57.38

PvKN4b 47.71 48.03 52.49 51.97 52.43 51.77 94.64

PvKN5a 49.38 50.78 51.44 50.95 46.45 47.12 49.19 44.30

PvKN5b 50.46 51.08 51.27 50.78 47.28 47.94 49.60 44.67 90.45

PvKN6a 47.12 47.84 49.82 49.12 46.07 46.43 47.64 44.96 45.05 45.92

PvKN6b 47.35 48.06 48.60 47.92 45.26 45.26 47.64 44.29 44.44 45.30 97.66

PvKN7a 48.70 48.34 48.92 48.38 48.69 49.06 48.70 45.90 47.89 47.72 83.86 84.21

PvKN7b 48.70 48.34 49.28 48.38 48.69 49.06 48.70 46.27 47.54 47.37 84.21 84.56 99.66

PvKN8a 29.96 29.63 30.19 30.08 28.52 28.63 31.98 29.39 26.52 27.50 30.45 30.83 32.06 32.06

PvKN8b 30.37 30.04 30.22 30.11 28.20 28.30 31.98 29.06 26.60 27.56 30.48 30.86 32.45 32.45 98.68

PvKN9a 32.84 31.87 31.97 30.77 30.11 29.74 32.03 31.25 34.04 34.15 33.96 33.33 33.08 32.71 60.69 60.82

PvKN9b 32.35 31.62 31.25 30.29 29.63 29.26 31.60 30.29 33.92 33.68 33.46 32.85 32.71 32.33 60.07 60.20 99.36

PvKN10a 31.37 30.63 32.96 31.48 31.32 31.32 34.53 33.58 32.03 31.80 36.57 37.04 34.60 34.98 61.43 61.15 89.49 88.96

PvKN10b 32.46 31.37 33.08 31.84 31.18 31.30 34.22 32.95 33.09 32.86 36.23 36.33 34.87 34.48 61.90 61.62 90.17 89.60 98.67

Amino acid sequences aligned with Muscle program (Edgar, 2004) and percent identity was calculated as the number of identical positions, including gaps divided by the length of the alignment.

**Supplementary Table 3** Features of switchgrass KNOX proteins compared to the maize ZmKN1 protein.

| **Gene** | **Amino acid identity with ZmKN1 (%)** | | | |
| --- | --- | --- | --- | --- |
|  | **HD domain**^b^ | **GSE**^c^ | **MEINOX**^d^ | **Entire sequence** |
| **Class I**^a^ | | | |  |
| PvKN1a | 98.4 | 100 | 92.8 | 90.7 |
| PvKN1b | 98.4 | 94.5 | 89.7 | 90.5 |
| PvKN2a | 88.7 | 25.8 | 62.5 | 54.0 |
| PvKN2b | 88.7 | 25.8 | 62.5 | 55.2 |
| PvKN3a | 82.3 | 12.5 | 56.7 | 47.1 |
| PvKN3b | 82.3 | 24.0 | 57.7 | 47.6 |
| PvKN4a | 80.7 | 13.8 | 55.7 | 53.5 |
| PvKN4b | 80.7 | 13.8 | 58.9 | 47.1 |
| PvKN5a | 82.3 | 30.3 | 50.5 | 47.8 |
| PvKN5b | 83.9 | 36.4 | 54.6 | 49.5 |
| PvKN6a | 74.2 | 16.7 | 51.6 | 48.8 |
| PvKN6b | 74.2 | 16.7 | 51.6 | 48.6 |
| PvKN7a | 75.8 | 8.1 | 48.4 | 49.6 |
| PvKN7b | 75.8 | 8.1 | 48.4 | 50.0 |
| **Class II**^a^ | | | |  |
| PvKN8a | 61.3 | 13.5 | 25.8 | 29.8 |
| PvKN8b | 61.3 | 13.5 | 25.8 | 29.1 |
| PvKN9a | 56.5 | 10.8 | 34.0 | 32.3 |
| PvKN9b | 56.5 | 10.8 | 33.0 | 32.2 |
| PvKN10a | 56.5 | 10.8 | 35.1 | 32.4 |
| PvKN10b | 56.5 | 10.8 | 35.1 | 32.1 |

1. Class I and II are phylogenetic classes of KNOX transcription factor family based on sequence similarity in the HD region, expression pattern and intron positions.
2. Homeodomain (HD) is a 60-63 amino acid sequence helix- turn -helix DNA-binding motif that characterizes KNOX genes family.
3. GSE is a relatively smaller less conserved domain found between the MEINOX and ELK domains
4. MEINOX is a conserved N-terminal domain shared between the MEIS genes in animals and KNOX genes in plants both belonging to a subclass of TALE (three amino acid loop extension) family of genes.

**Supplementary Table 4** List of the locus names and/or GenBank accession numbers of the sequences used in this study.

| **Gene name** | **Locus name/accession number** | **Species** |
| --- | --- | --- |
| PvKN1a | Pavir.Ca01130 | *Panicum virgatum* |
| PvKN1b | Pavir.J13005 | *P. virgatum* |
| PvKN2a | Pavir.Ea01271 | *P. virgatum* |
| PvKN2b | Pavir.Eb02382 | *P. virgatum* |
| PvKN3a | Pavir.J27068 | *P. virgatum* |
| PvKN3b | Pavir.Eb03210 | *P. virgatum* |
| PvKN4a | Pavir.Ca01636 | *P. virgatum* |
| PvKN4b | Pavir.J11479 | *P. virgatum* |
| PvKN5a | Pavir.Ba04002 | *P. virgatum* |
| PvKN5b | Pavir.Bb00108 | *P. virgatum* |
| PvKN6a | Pavir.Ga01287 | *P. virgatum* |
| PvKN6b | Pavir.Gb01218 | *P. virgatum* |
| PvKN7a | Pavir.Ea00180 | *P. virgatum* |
| PvKN7b | Pavir.J04662 | *P. virgatum* |
| PvKN8a | Pavir.J34867 | *P. virgatum* |
| PvKN8b | Pavir.Ca00918 | *P. virgatum* |
| PvKN9a | KanlCTG23388 (Switchgrass unitranscript ID) | *P. virgatum* |
| PvKN9b | Pavir.J11300 | *P. virgatum* |
| PvKN10a | Pavir.Da02356 | *P. virgatum* |
| PvKN10b | Pavir.J38727 | *P. virgatum* |
| WKNOX1b | AF224499_1 | *Triticum aestivum* |
| WRS1 | BAH03543 | *T. aestivum* |
| WKNOX1a | AF224498_1 | *T. aestivum* |
| HvKNOX3 | BAK07974 | *Hordeum vulgare* |
| HvKN1 | AAQ11882 | *H. vulgare* |
| SbKN1 | ABC71525 | *Sorghum bicolor* |
| SbKN2 | Sb10g025440 | *S. bicolor* |

**Supplementary Table 4** Continued.

| **Gene name** | **Locus name/accession number** | **Species** |
| --- | --- | --- |
| SbKN3 | XP_002451671 | *S. bicolor* |
| SbKN4 | Sobic.001G526200 | *S. bicolor* |
| OsHOS3 | BAA77817 | *Oryza sativa* ssp*. Japonica* |
| OSH1 | ABF98653/ LOC_Os03g51690 | *O. sativa* ssp*. J.* |
| OSH45 | KNOSD_ORYSJ/LOC_Os08g19650 | *O. sativa* ssp*. J.* |
| HOS66 | KNOS3_ORYSJ/LOC_Os03g03164 | *O. sativa* ssp*. J.* |
| HOS58 | KNOS2_ORYSJ/LOC_Os02g08544 | *O. sativa* ssp*. J.* |
| HOS59 | KNOSB_ORYSJ/LOC_Os06g43860 | *O. sativa* ssp*. J.* |
| ZmRS1 | NP_001149651 | *Zea mays* |
| ZmKN3 | XP_008659775 | *Z. mays* |
| ZmKN1 | NP_001105436 | *Z. mays* |
| ZmKN2 | XP_008660553 | *Z. mays* |
| ZmKN4 | AFW66300 | *Z. mays* |
| ZmKN6 | NP_001150419 | *Z. mays* |
| ZmKN5 | AFW70462 | *Z. mays* |
| SiKN1 | ABC71528 | *Setaria italica* |
| SiKN5 | XP_004985913 | *S. italica* |
| SiKN3 | XP_004951667 | *S. italica* |
| SiKN4 | XP_004973169 | *S. italica* |
| SiKN2 | XP_004965737 | *S. italica* |
| BdKN1 | XP_003558936 | *Brachypodium distachyon* |
| BdKN2 | XP_003563314 | *B. distachyon* |
| BdKN4 | XP_003573667 | *B. distachyon* |
| BdKN3 | XP_003570788 | *B. distachyon* |
| AtqKNOX1 | ADN43388 | *Agave tequilana* |
| PmKN1 | ABC71526 | *Panicum miliaceum* |
| KNAT1 | AT4G08150/NP_192555 | *Arabidopsis thaliana* |

**Supplementary Table 4** Continued.

| **Gene name** | **Locus name/accession number** | **Species** |
| --- | --- | --- |
| KNAT2 | NP_177208/ AT1G70510 | *A. thaliana* |
| KNAT3 | AT5G25220/ NP_001031938 | *A. thaliana* |
| KNAT4 | AT5G11060/ NP_196667 | *A. thaliana* |
| KNAT5 | NP_194932/ AT4G32040 | *A. thaliana* |
| KNAT6 | NP_850951/ AT1G23380 | *A. thaliana* |
| KNAT7 | NP_564805/ AT1G62990 | *A. thaliana* |
| STM | NP_176426/AT1G62360 | *A. thaliana* |
| PpKNOPE1 | ABD52723 | *Prunus persica* |
| PpKNOPE2 | ABO28750 | *P. persica* |
| PpKNOPE2.1 | JQ038131 | *P. persica* |
| PpKNOPE3 | ACJ71731 | *P. persica* |
| PpKNOPE4 | ABO26062 | *P. persica* |
| PpKNOPE6 | ADC35598.1 | *P. persica* |
| PpKNOPE7 | JQ038132 | *P. persica* |
| ChBP | ABG66654 | *Cardamine hirsuta* |
| BrBP | ACS28249 | *Brassica rapa* |
| BoBP | ACS28250 | *Brassica oleracea* |
| BnBP | ACR83812 | *Brassica napus* |
| MtKNOX | ABO33479 | *Medicago truncatula* |
| KNAP2 | KNAP2_MALDO | *Malus domestica* |
| IbKN3 | BAF93480 | *Ipomoea batatas* |
| IbKN2 | BAF93479 | *I. batatas* |
| SlKN1 | NP_001233807 | *Solanum lycopersicum* |
| NtKN2 | AAQ11889 | *Nicotiana tabacum* |
| NtKN3 | AAQ11890 | *N. tabacum* |
| HaKN2 | AAM28232 | *Helianthus annuus* |
| LjKN2 | AAX21346 | *Lotus japonicus* |
| ARK1 | AAV28488 | *Populus tremula x Populus alba* |
| PtKN1 | XP_002301134 | *Populus trichocarpa* |


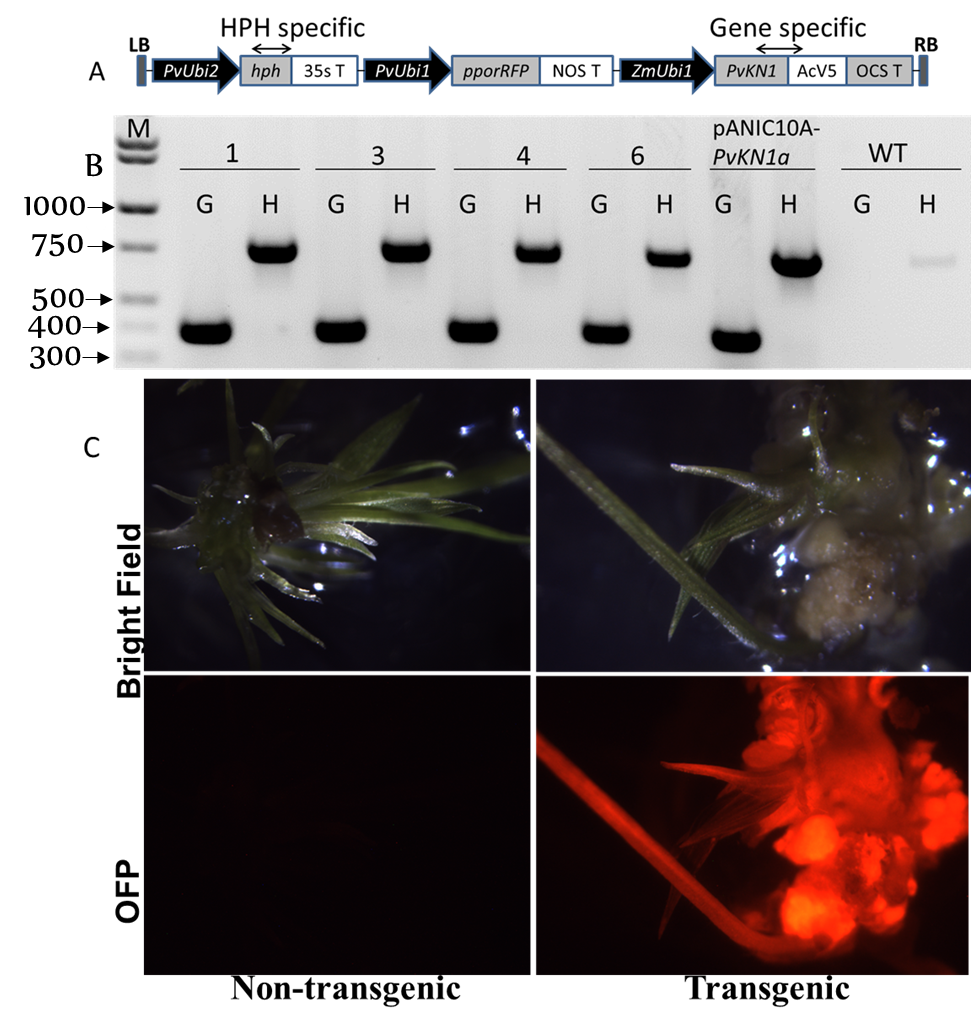


**Supplementary Figure 1** Molecular characterization of transgenic switchgrass plants overexpressing the *PvKN1a* gene. A) The T-DNA from pANIC10A vector used for overexpression of *PvKN1a* in transgenic switchgrass. pANIC10A vector has switchgrass ubiquitin 1(PvUbi1) and 2 (PvUbi2) promoters driving the expression of *pporRFP* (orange flourescent protein) and hygromycin phosphotransferase (HPH) gene, respectivley, and the gene of interest (PvKN1) is driven by maize ubiquitin (ZmUbi1) promoter. B) Genomic PCR for assaying transgene (G) insertion and the hygromycin-resistance (H) genes in putative transgenic lines (1, 3, 4 and 6). Hi-Lo DNA marker sizes were indicated by arrows. C) Orange fluorescence protein (*pporRFP*; OFP) visualization in transgenic plants compared to the non-transgenic control.

**
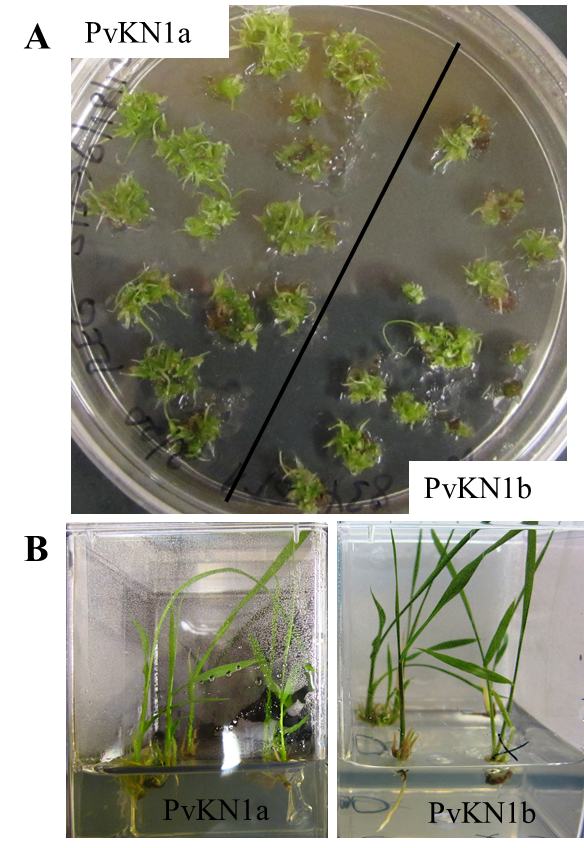
**

**Supplementary Figure 2** Comparison of morphological phenotypes in transgenic switchgrass overexpressing *PvKN1a* and *PvKN1b*. Transformants overexpressing *PvKN1a* and *PvKN1b* after shoot regeneration (A) and 9-weeks after transferred to rooting media.


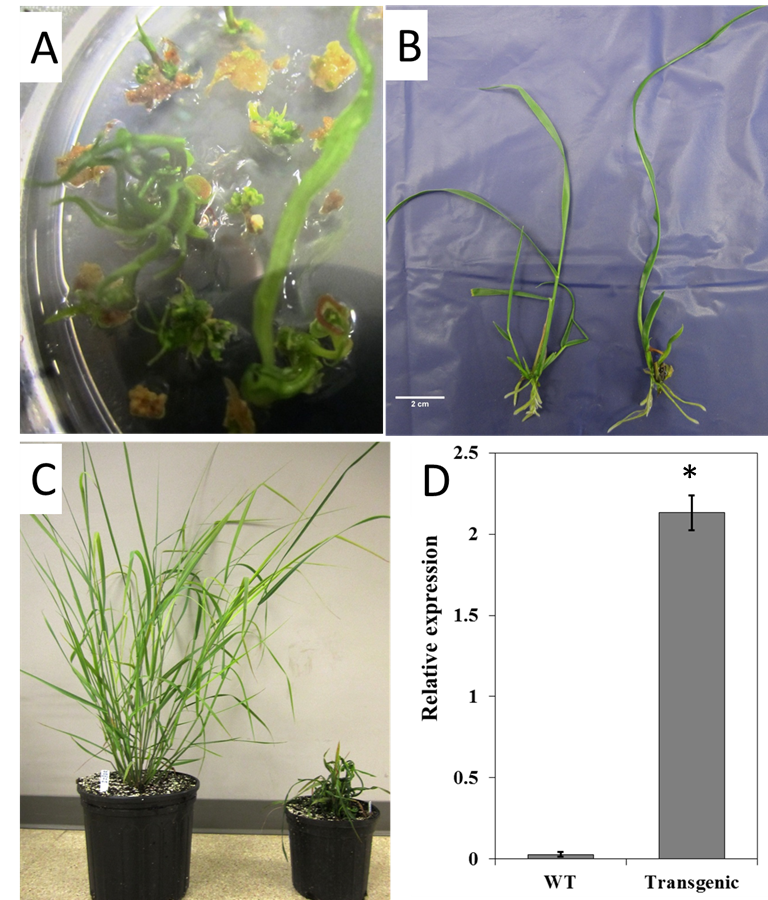


**Supplementary Figure 3** Transgenic switchgrass (SA37) plants overexpressing *PvKN1a* showing abnormal phenotypes throughout various stages of development. A) Transformants at early developmental stage B) Rooted seedlings of non-transgenic (left) and transgenic (right) lines C) Mature non-transgenic (left) and transgenic (right) lines at about 4 months after transferred to soil. D) Relative transcript levels of *PvKN1 i*n transgenic and non-transgenic control (WT) lines. The relative levels of transcripts were normalized to ubiquitin (UBQ). Asterisks indicate significant differences from non-transgenic control plants at *P* ≤ 0.05 as determined by PROC TTEST procedure using SAS software (SAS Institute Inc.). Bars represent mean values of 3 replicates ± standard error.


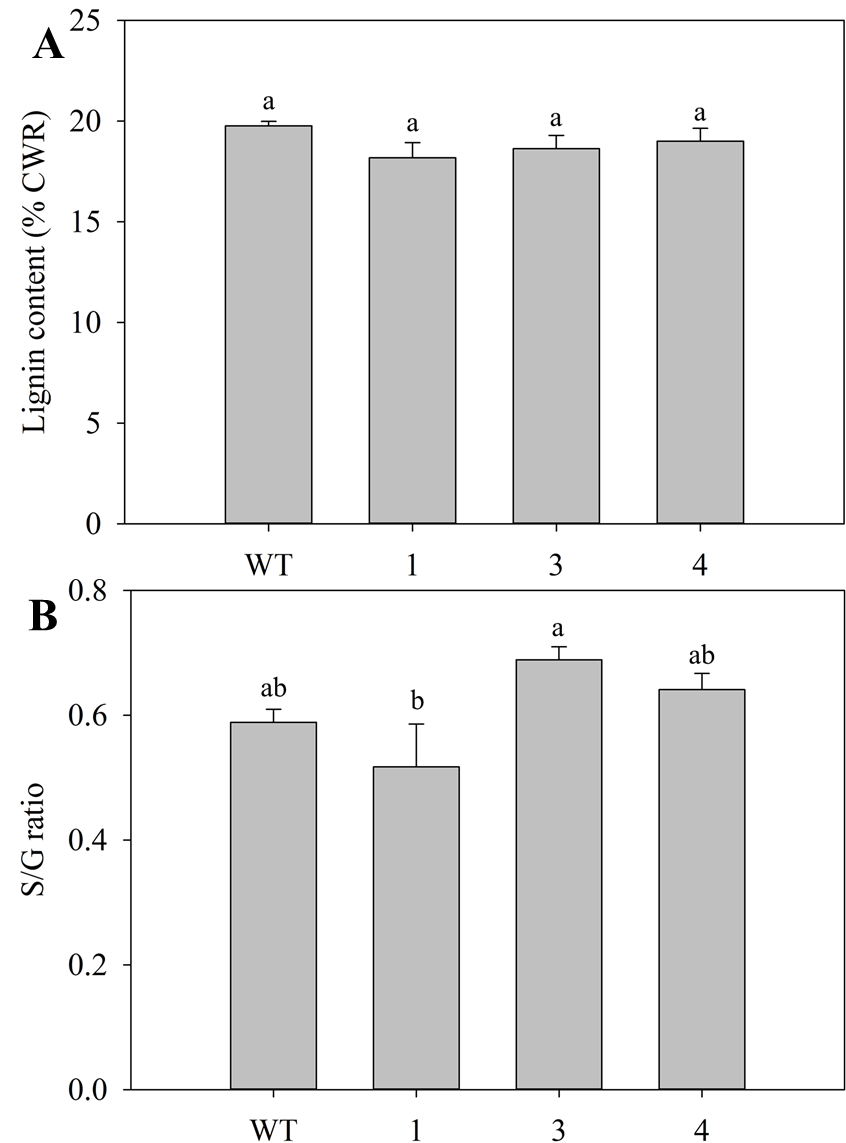


**Supplementary Figure 4** Lignin content (A) and S/G ratio (B) of transgenic and non-transgenic (WT) switchgrass lines as determined via pyrolysis- molecular beam mass spectrometry. All data represent the average of the replicates ± standard deviation. Bars represented by different letters are significantly different at *P* ≤ 0.05 as tested by LSD method with SAS software (SAS Institute Inc.). CWR, cell wall residues.


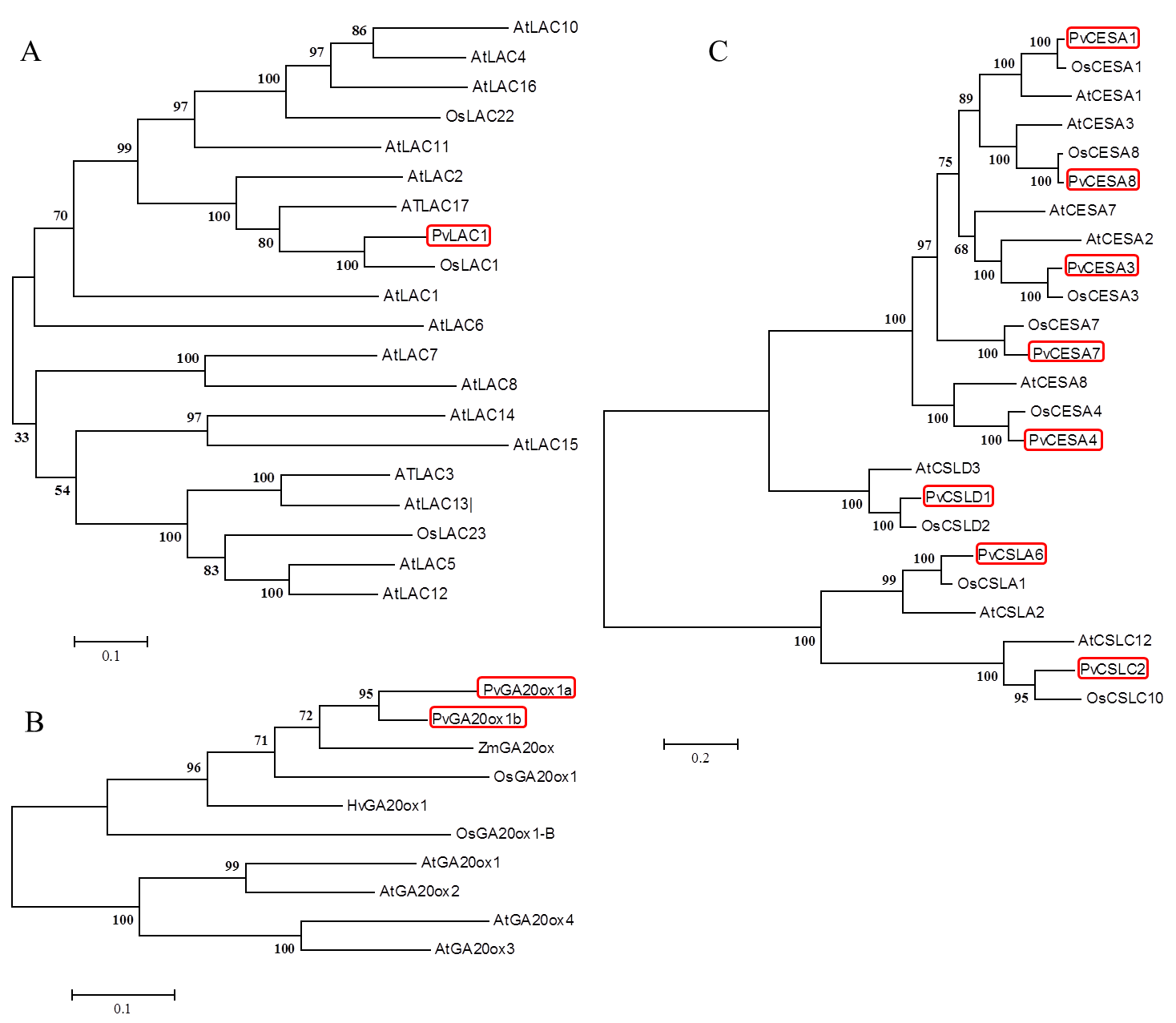


**Supplementary Figure 5** Dendrogram showing the relatedness of the PvKN1 target genes [PvLAC1 (A), GA20ox1 (B) and cellulose and hemicellulose biosynthetic genes (C)] indicated in figure 7 with the known homologs in monocots and *Arabidopsis*. Cluster analysis was performed using the deduced amino acid sequences from switchgrass along with already-characterized proteins from monocots and *Arabidopsis*. The sequences were aligned using MUSCLE program (Edgar, 2004). The tree was constructed by maximum likelihood procedure using MEGA6.0 program (Tamura et al., 2013). Analysis using 1000 bootstrap replicates was performed. The scale bar shows 0.1 or 0.2 amino acid substitutions per site. The unitranscript/locus IDs of switchgrass genes are listed in Table S4. Locus IDs of the genes from barley, rice, maize and *Arabidopsis* are: HvGA20ox1 (AAT49058.1), ZmGA20ox1 (NP_001241783.1), OsGA20ox1 (LOC_Os03g63970.1), OsGA20ox1-B (LOC_Os07g07420.1), OsLAC1 (LOC_Os01g62480.1), OsLAC22 (LOC_Os11g48060.1), OsLAC23 (LOC_Os12g01730.1), OsCSLA1 (LOC_Os02g09930.1), OsCESA1 (LOC_Os05g08370.1), OsCESA3 (LOC_Os07g24190.1), OsCESA7 (Os10g32980.1), OsCESA8 (LOC_Os07g10770.1), OsCESA4 (Os01g54620.1), OsCSLD2 (LOC_Os06g02180.1), OsCSLC10 (LOC_Os07g03260.1), AtGA20ox1 (AT4G25420), AtGA20ox2 (AT5G51810), AtGA20ox3 (AT5G07200), AtGA20ox4 (AT1G60980), AtCESA1 (AT4G32410), AtCESA2 (AT4G39350.1), AtCESA3 (AT5G05170), AtCESA7 (AT5G17420.1), AtCESA8 (AT4G18780), AtCSLA2 (AT5G22740), AtCSLC12 (AT4G07960), AtCSLD3 (AT3G03050), AtLAC1 (AT1G18140), AtLAC2 (AT2G29130), AtLAC3 (AT2G30210), AtLAC4 (AT2G38080), AtLAC5 (AT2G40370), AtLAC6 (AT2G46570), AtLAC7 (AT3G09220), AtLAC8 (AT5G01040), AtLAC10 (AT5G01190), AtLAC11 (AT5G03260), AtLAC12 (AT5G05390), AtLAC13 (AT5G07130), AtLAC14 (AT5G09360), AtLAC15 (AT5G48100), AtLAC16 (AT5G58910), AtLAC17 (AT5G60020).
